# DUSP1 mediates BCG induced apoptosis and inflammatory response in THP-1 cells via MAPKs/NF-κB signaling pathway

**DOI:** 10.1038/s41598-023-29900-6

**Published:** 2023-02-14

**Authors:** Zhanyou Liu, Jianhong Wang, Fan Dai, Dongtao Zhang, Wu Li

**Affiliations:** 1 Key Lab of Ministry of Education for Protection and Utilization of Special Biological Resources in Western China, Yinchuan, 750021 Ningxia China; 2 School of Life Sciences, Ningxia University, 539 W. Helanshan Road, Yinchuan, 750021 Ningxia China

**Keywords:** Immunology, Microbiology

## Abstract

Tuberculosis (TB) is a zoonotic infectious disease caused by *Mycobacterium tuberculosis* (Mtb). Apoptosis and necrosis caused by the interaction between the host and the pathogen, as well as the host’s inflammatory response, play an important role in the pathogenesis of TB. Dual-specificity phosphatase 1 (DUSP1) plays a vital role in regulating the host immune responses. However, the role of DUSP1 in the regulation of THP-1 macrophage apoptosis induced by attenuated *Mycobacterium bovis* Bacillus Calmette-Guérin (BCG) infection remains unclear. In the present study, we report that infection with BCG significantly induces macrophage apoptosis and induces the production of DUSP1, TNF-α and IL-1β. DUSP1 knockdown significantly inhibited BCG-induced macrophage apoptosis and activation of MAPKs/NF-κB signaling pathway. In addition, DUSP1 knockdown suppressed BCG-induced inflammation in vivo. Taken together, this study demonstrates that DUSP1, as a regulator of MAPKs/NF-κB signaling pathway, plays a novel role in BCG-induced macrophage apoptosis and inflammatory response.

## Introduction

According to the World Health Organization (WHO), tuberculosis (TB), caused by the infection of *Mycobacterium tuberculosis* (Mtb), remains one of the world's deadliest infectious diseases, with approximately 1.49 million deaths worldwide in 2020^1^. The pathogenesis of tuberculosis and the immune responses underlying protection are poorly understood. In addition, the continued spread of drug-resistant, multi-drug resistant and even extremely drug-resistant TB, as well as the co-infection of Mtb with Human Immunodeficiency Virus (HIV), pose serious challenges to TB prevention and control^2^. Moreover, the global epidemic of COVID-19 has made it more difficult to effectively prevent and control the spread of TB^1^. Therefore, the discovery of novel molecular targets is urgently needed for developing new anti-TB drugs.

Both innate and adaptive immune responses are involved in host defense against Mtb, and macrophages are considered to be the key effector cells of the innate immune system. Macrophages can phagocytose bacteria and secrete both pro-inflammatory and antimicrobial mediators to kill the invading Mtb^3^. Macrophage apoptosis induced by Mtb infection can also kill the invading bacteria and inhibit their dissemination in vivo^4^. However, Mtb can survive inside macrophages post-phagocytosis using various mechanisms, including inhibition of apoptosis and oxidative stress^5^. Apoptotic pathways can be classified as endogenous, exogenous, and endoplasmic reticulum pathways^6^. Regardless of the pathway, cells undergoing apoptosis eventually degrade into multiple apoptotic vesicles by the action of caspase-3 and are engulfed by surrounding macrophages, causing the cells to lyse^7^. Apoptosis by any mechanism is an important way for macrophages to resist bacterial infection.

Mitogen-activated protein kinases (MAPKs) are key regulators of cellular responses and signal transduction^8^. MAPKs mainly include extracellular signal-regulated kinase 1 and 2 (ERK1/2), p38 MAPK and c-Jun N-terminal kinase (JNK). Studies have shown that MAPKs play an important role in cell proliferation and apoptosis, and are also key regulators of mitochondrial division and the mitochondrial pathway of apoptosis^8,9^. Dual-specificity phosphatases (DUSPs) are members of the protein tyrosine phosphatase (PTP) superfamily and can be divided into two major groups, typical and atypical DUSP^10^. Typical DUSPs mainly function to dephosphorylate MAPKs, while for atypical DUSPs, the relationship to MAPKs activation is complex and still unknown^11^. DUSP1, also known as mitogen-activated protein kinase phosphatase-1 (MKP-1), can dephosphorylate MAPKs or interfere with effector molecules that bind to MAPKs, thereby inhibiting MAPKs activity^12^. The nuclear factor kappa B (NF-κB) signaling pathway plays a critical role in natural immunity and inflammatory responses^13^. Inhibition of MAPKs/NF-κB signaling pathway is thought to be effective in attenuating Mtb-induced inflammatory responses^14^. Overexpression of DUSP1 attenuates apoptosis and inflammation by inhibiting MAPKs/NF-κB signaling pathway activation in disease models such as myocardial injury^15^ and colonic inflammation^16^. However, whether DUSP1 has any effect on BCG-induced macrophage apoptosis and MAPKs/NF-κB signaling in the inflammatory responses is still unclear.

The objective of this study was therefore to investigate the function of DUSP1 in macrophages after infection with Mtb. We identified the molecular mechanisms underlying BCG-induced macrophage apoptosis and inflammation, and found that DUSP1 interference inhibited BCG-induced THP-1 apoptosis and activation of MAPKs/NF-κB signaling in THP-1 macrophages. From a mechanistic perspective, DUSP1 interference inhibited MAPKs/NF-κB activation, finally inhibited macrophage apoptosis and the production of TNF-α and IL-1β. In vivo studies of BCG infection of DUSP1 knockdown mice further confirmed the proposed mechanism. These results illustrate an important role of DUSP1 in BCG-induced macrophage apoptosis and inflammatory responses, suggesting DUSP1 as a novel target for anti-TB drug discovery that warrants for further investigation.

## Results

### BCG infection induces apoptosis in THP-1 cells and promotes the expression of DUSP1 and pro-inflammatory cytokines

**Figure 1:**
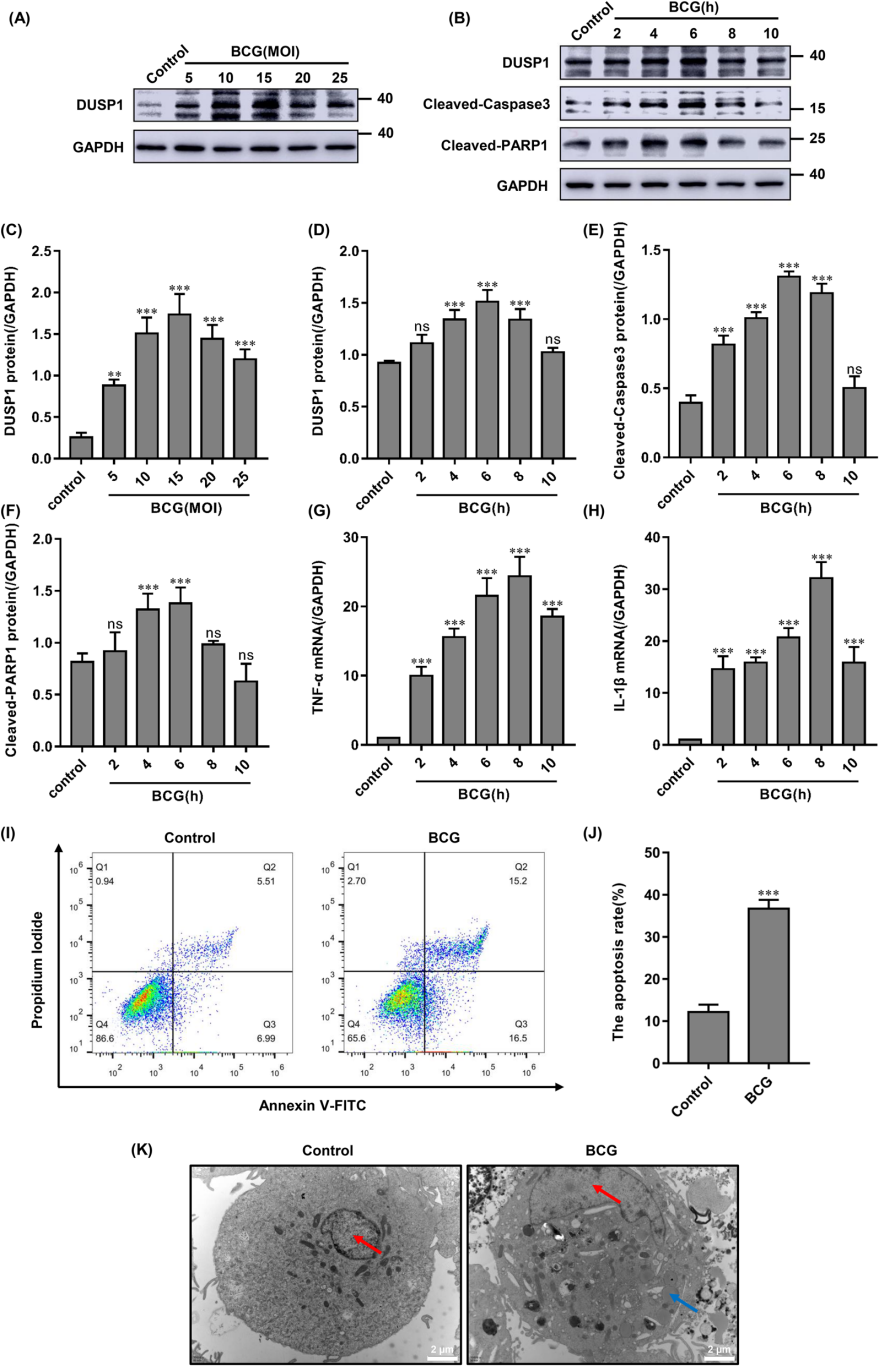
BCG infection induces apoptosis in THP-1 cells and promotes the expression of DUSP1 and pro-inflammatory cytokines. (**A**, **C**) DUSP1 protein expression detected by Western blotting after 6 h of BCG infection of THP-1 cells at different MOI. (**B**, **D**–**F**) DUSP1, Cleaved-Caspase3 and Cleaved-PARP1 protein expression detected by Western blotting after BCG (MOI = 10) infection at different time points. (**G**, **H**) Gene expression of TNF-α and IL-1β detected by RT-qPCR after different times of BCG infection (MOI = 10). (**I**, **J**) After BCG infection of THP-1 cells for 6 h, the apoptosis rate was detected by flow cytometry. (**K**) After BCG infection of THP-1 cells for 6 h, the ultrastructure of macrophages was observed by transmission electron microscopy (10,000 ×), the blue arrow denotes the cytoplasmic vacuole of apoptotic cells, while the red arrow indicates the nucleus. Scale bar, 2 μm. All data are represented as mean ± SD (n = 3). (*p < 0.05, **p < 0.01, ***p < 0.001).

Studies have shown that DUSP1 plays an important role in regulation of apoptosis and inflammatory response^17,18^. In this study we examine whether BCG infection upregulates DUSP1 expression and whether DUSP1 is involved in BCG-induced apoptosis and inflammatory response. We showed via Western blotting that DUSP1 expression was significantly upregulated after treatment of THP-1 cells with BCG at different multiplicities of infection (MOI) at different time points post-infection (Fig. 1A–D). Expression levels of pro-apoptotic proteins Cleaved-Caspase3 and Cleaved-PARP1 were also significantly upregulated (Fig. 1B,E,F), reaching their highest levels at 6 h post-BCG infection and then gradually returning to baseline levels. Given that TNF-α and IL-1β-induced activation of MAPKs and NF-κB plays an important role in the inflammatory response, we measured the release of pro-inflammatory cytokines TNF-α and IL-1β in THP-1 cells by RT-qPCR. After infection of THP-1 with BCG (MOI = 10) for various time intervals, TNF-α and IL-1β transcripts were significantly upregulated in a time-dependent manner (Fig. 1G,H). Flow cytometric analysis showed that BCG infection significantly increased the apoptosis rate of macrophages (Fig. 1I,J). Transmission electron microscopy observation showed that BCG-infected cells exhibited a variety of apoptotic features, specifically, star-moon shaped nuclei, ruptured cell membranes, formation of many vacuolar structures, and incomplete organelle structures, whereas healthy cells had intact cell membranes and clearly visible organelle structures (Fig. 1K). These results suggested that BCG infection induced macrophage apoptosis and promoted the expression of DUSP1 and pro-inflammatory cytokines. For subsequent experiments, 6 h of BCG infection (MOI = 10) was chosen as the time of infection.

### Establishment of a model of THP-1 cell infection by siRNA-DUSP1 combined with BCG

**Figure 2:**
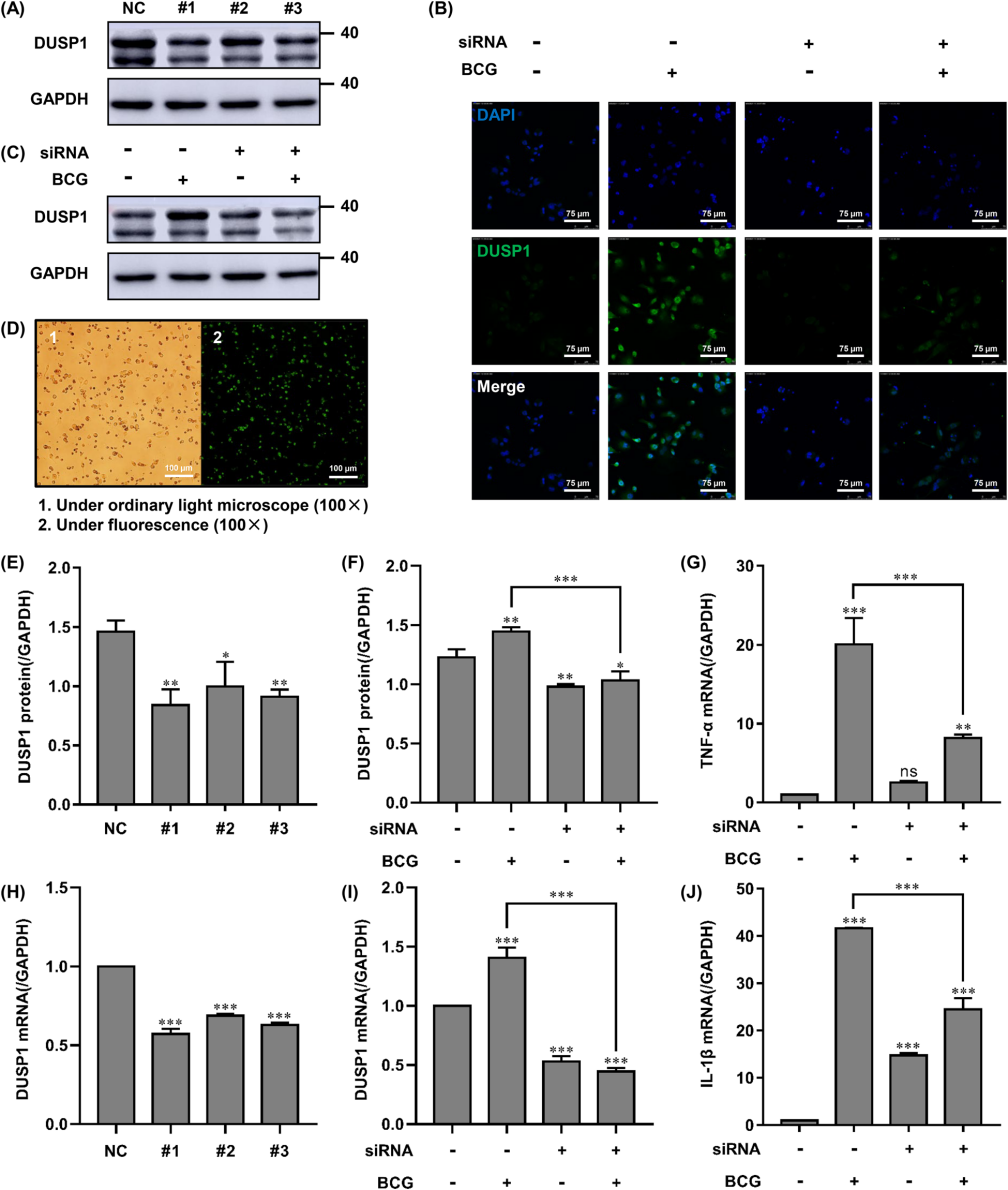
Effect of DUSP1 knockdown on DUSP1, TNF-α and IL-1β in BCG-induced THP-1 cells. (**A**, **E**, **H**) THP-1 cells were transfected with three different targeting siRNA-DUSP1 for 24 h, and knockdown was verified by Western blotting and RT-qPCR. (**B**, **C**, **F**, **I**) THP-1 cells were transfected with siRNA-NC (negative control) or siRNA-DUSP1 for 24 h and then infected with BCG for 6 h, and the knockdown effect was verified by immunofluorescence, Western blotting and RT-qPCR to verify the effect of DUSP1 knockdown. Scale bar, 75 μm. (**D**) Observation of siRNA-DUSP1 transfection efficiency by fluorescence microscopy. Scale bar, 150 μm. (**G**, **J**) THP-1 cells were transfected with siRNA-NC (negative control) or siRNA-DUSP1 for 24 h and then infected with BCG for 6 h. Expression of TNF-α and IL-1β was detected by RT-qPCR. All data are represented as mean ± SD (n = 3). (*p < 0.05, **p < 0.01, ***p < 0.001).

Given that BCG infection can upregulate DUSP1 expression, we hypothesized that DUSP1 may be involved in BCG-induced apoptosis and inflammatory responses. We designed and synthesized three small interfering RNA sequences based on the DUSP1 mRNA sequence, transfected siRNA-DUSP1 into post-adherent THP-1 cells, and observed the effect by fluorescence microscopy after 24 h (Fig. 2D). We then detected the efficiency of knockdown of the three small interfering RNAs by RT-qPCR and Western blotting. The results showed that compared to the negative control transfection group, small interfering RNA #1 was the most efficient at knockdown, and both DUSP1 protein and mRNA expression levels were highly down-regulated (Fig. 2A,E,H). To further verify the knockdown effect of siRNA-DUSP1, macrophages were transfected with siRNA-DUSP1 followed by infection with BCG (MOI = 10) 24 h post-transfection, and cultured for 6 h. The knockdown effect of siRNA-DUSP1 was measured by RT-qPCR and Western blotting. Both DUSP1 protein and mRNA expression were significantly downregulated in the siRNA + BCG group as compared with the BCG-only group (Fig. 2C,F,I). The immunofluorescence assay supported the data obtained by Western blotting and RT-qPCR. These results also demonstrated that siRNA-DUSP1 could reduce the number of DUSP1-positive cells after BCG infection (Fig. 2B). Taken together, these findings indicated that DUSP1 knockdown combined with BCG-infection model was successful. In addition, the release of TNF-α and IL-1β induced by BCG (MOI = 10) infection was reversed by siRNA-DUSP1 transfection (Fig. 2G,J), indicating that DUSP1 knockdown inhibited the BCG-induced inflammatory response in THP-1 cells.

### Knockdown of DUSP1 reduces BCG-induced apoptosis in macrophages

**Figure 3:**
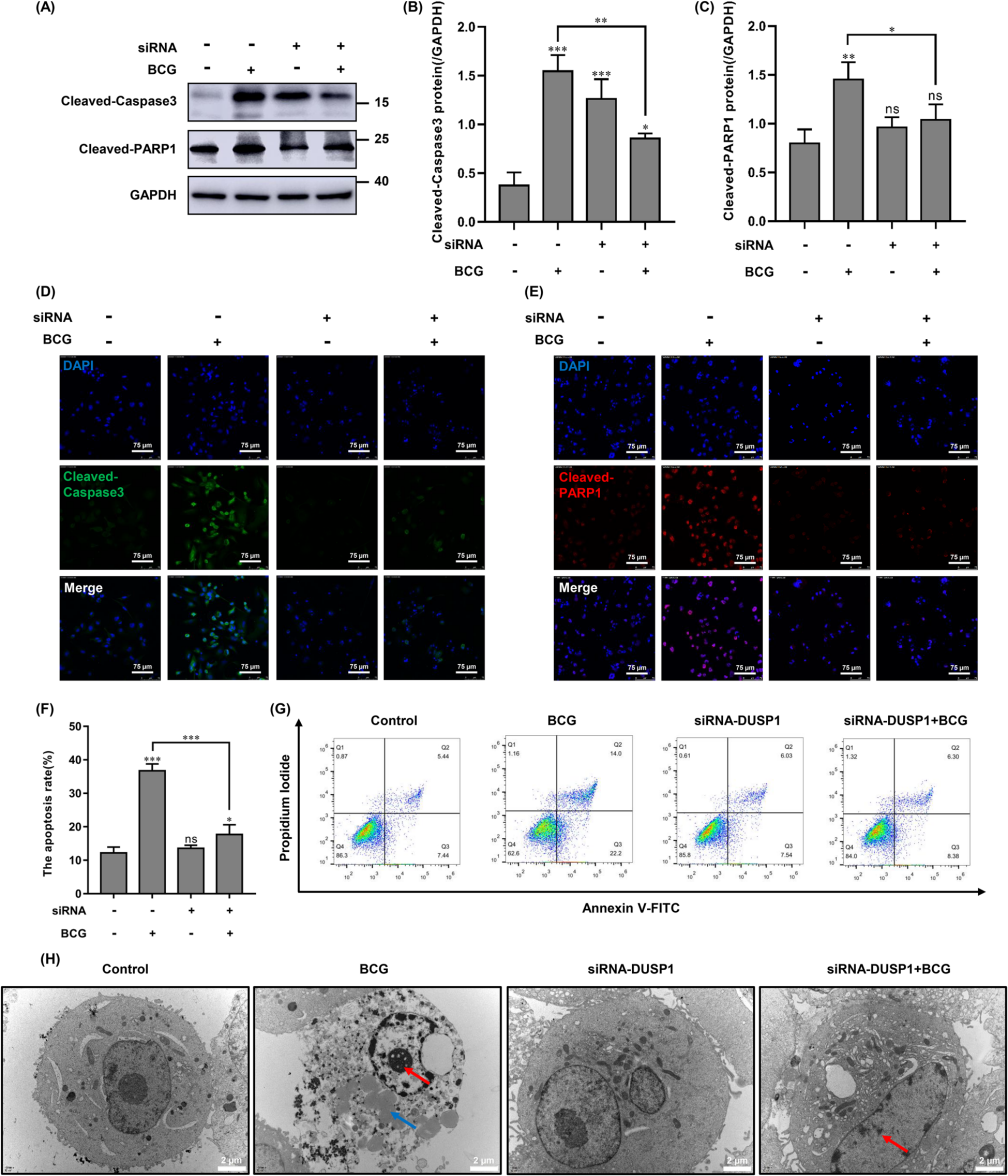
Effect of knockdown of DUSP1 on BCG-induced apoptosis in macrophages. (**A**–**C**) THP-1 cells were transfected with siRNA-NC (negative control) or siRNA-DUSP1 for 24 h and then infected with BCG and cultured for 6 h. Cleaved-Caspase3 and Cleaved-PARP1 protein expression was detected by western blotting. (**D**, **E**) Knockdown of DUSP1 combined with BCG infection were detected by immunofluorescence technique for Cleaved-Caspase3 and Cleaved-PARP1 expression levels. Scale bar, 75 μm. (**F**, **G**) THP-1 cells were transfected with siRNA-NC (negative control) or siRNA-DUSP1 for 24 h and then infected with BCG and cultured for 6 h, apoptosis was determined by Annexin V/PI staining using FCS analysis. (**H**) THP-1 cells were transfected with siRNA-NC or siRNA-DUSP1 for 24 h and then infected with BCG and cultured for 6 h, the morphological effects of DUSP1 on apoptosis of BCG-infected THP-1 cells were observed by transmission electron microscopy, the blue arrow denotes the cytoplasmic vacuole of apoptotic cells, while the red arrow indicates the nucleus. Scale bar, 2 μm. All data are represented as mean ± SD (n = 3). (*p < 0.05, **p < 0.01, ***p < 0.001).

To investigate whether DUSP1 plays a role in regulating BCG-induced macrophage apoptosis, we transfected siRNA-DUSP1 into THP-1 cells and then infected with BCG, followed by culturing for 6 h. The expression of cleaved-Caspase3 and cleaved-PARP1 was detected by Western blotting, and the results showed that expression of both cleaved-Caspase3 and cleaved-PARP1 was significantly downregulated in the siRNA + BCG group compared to the BCG-alone infected group (Fig. 3A–C). Next, we examined the expression levels of cleaved-Caspase3 and Cleaved-PARP1 by immunofluorescence and found that both proteins were significantly downregulated in the siRNA + BCG group, in agreement with the Western blotting results (Fig. 3D,E). These data indicate that knockdown of DUSP1 reduces BCG-induced apoptosis of macrophages.

To further verify whether knockdown of DUSP1 can modulate BCG-induced macrophage apoptosis, we detected apoptotic cells using Annexin V-FITC/PI double staining. These results showed that BCG infection significantly increased apoptosis of macrophages, but when BCG infection was combined with siRNA-DUSP1 treatment, apoptosis could be reduced significantly (Fig. 3F,G). At the ultrastructural level (Fig. 3H), siRNA-DUSP1 attenuated nuclear division, cytoplasmic cohesion and vesicle reduction in BCG-infected THP-1 cells, suggesting that DUSP1 was able to regulate BCG-induced macrophage apoptosis.

### Knockdown of DUSP1 regulates BCG-induced apoptosis in macrophages via the endogenous apoptotic pathway

**Figure 4:**
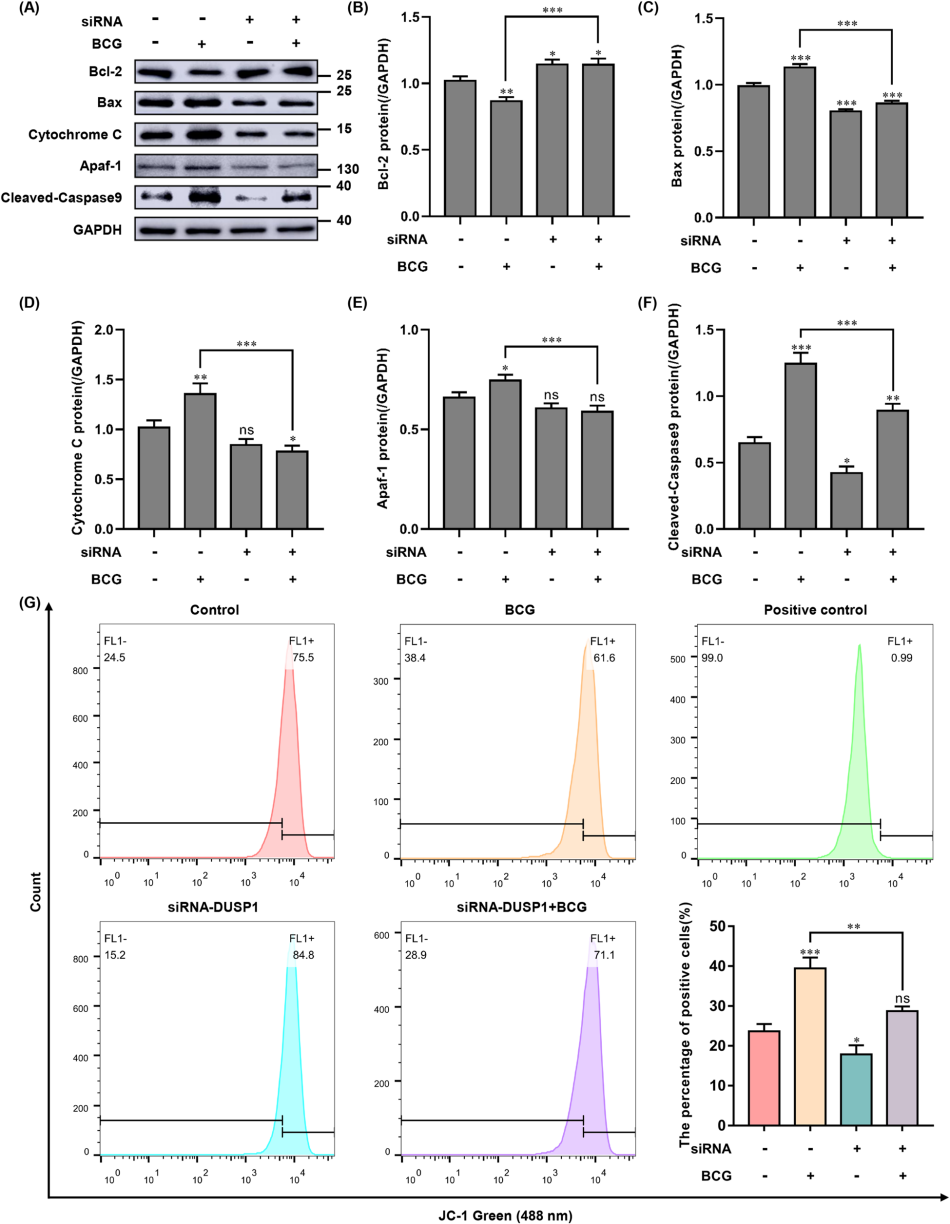
Effect of knockdown of DUSP1 on the expression of key proteins of BCG-induced endogenous apoptosis pathway in macrophages. (**A**) THP-1 cells were transfected with siRNA-NC (negative control) or siRNA-DUSP1 for 24 h, and then infected with BCG and cultured for 6 h. The expression levels of endogenous apoptotic pathway key proteins Bcl-2, Bax, cytochrome C, Apaf-1, cleaved-Caspase9 were detected using Western blotting. (**B**–**F**) Endogenous apoptotic pathway key results of grey-scale analysis of protein expression. (**G**) THP-1 cells were transfected with siRNA-NC (negative control) or siRNA-DUSP1 for 24 h, and then infected with BCG and cultured for 6 h. Mitochondrial membrane potential was detected by flow cytometry. All data are represented as mean ± SD (n = 3). (*p < 0.05, **p < 0.01, ***p < 0.001).

Apoptosis is initiated as a result of a stress response to an appropriate stimulus. BCG infection causes cytochrome C to be released from the mitochondria, where it binds to the apoptotic factor Apaf-1 in the presence of dATP to form a multimer, leading to the activation of Caspase-9, which in turn cleaves Caspase3 and PARP1, thereby triggering cell apoptosis^19^. The Bcl-2 family has many members, such as Bax, Bcl-2 and Bad, which each play important roles in the regulation of apoptosis^20^. Bcl-2 is located in the nuclear membrane, mitochondria and endoplasmic reticulum, and can prevent apoptosis by inhibiting the release of cytochrome C from mitochondria, while Bax and Bad can promote apoptosis^21^. To investigate the effect of DUSP1 knockdown on BCG-induced endogenous apoptosis in macrophages, we detected Bax, Bcl-2, cytochrome C, Apaf-1 and cleaved-Caspase-9 expression levels by Western blotting. The results showed that the expression of cleaved-Caspase9, Apaf-1, Bax and cytochrome C was significantly downregulated in the siRNA + BCG group compared with the BCG-alone group, and the expression of Bcl-2, an apoptosis inhibitor, was significantly upregulated (Fig. 4A–F). These results suggest that DUSP1 knockdown reduces BCG-induced macrophage apoptosis via the endogenous apoptotic pathway.

Changes in mitochondrial membrane potential (MMP) reflect the permeability of the mitochondrial membrane and are also considered to be one of the typical features of apoptosis onset. JC-1 is a fluorescent probe used to detect mitochondrial membrane potential, and therefore we stained THP-1 cells with JC-1 and assessed the mitochondrial integrity of cells by flow cytometry. The results showed a significant increase in mitochondrial depolarization of macrophages following BCG infection, which was alleviated by knockdown of DUSP1 (Fig. 4G).

### Knockdown of DUSP1 regulates BCG-infected macrophages via MAPKs/NF-κB signaling pathway

**Figure 5:**
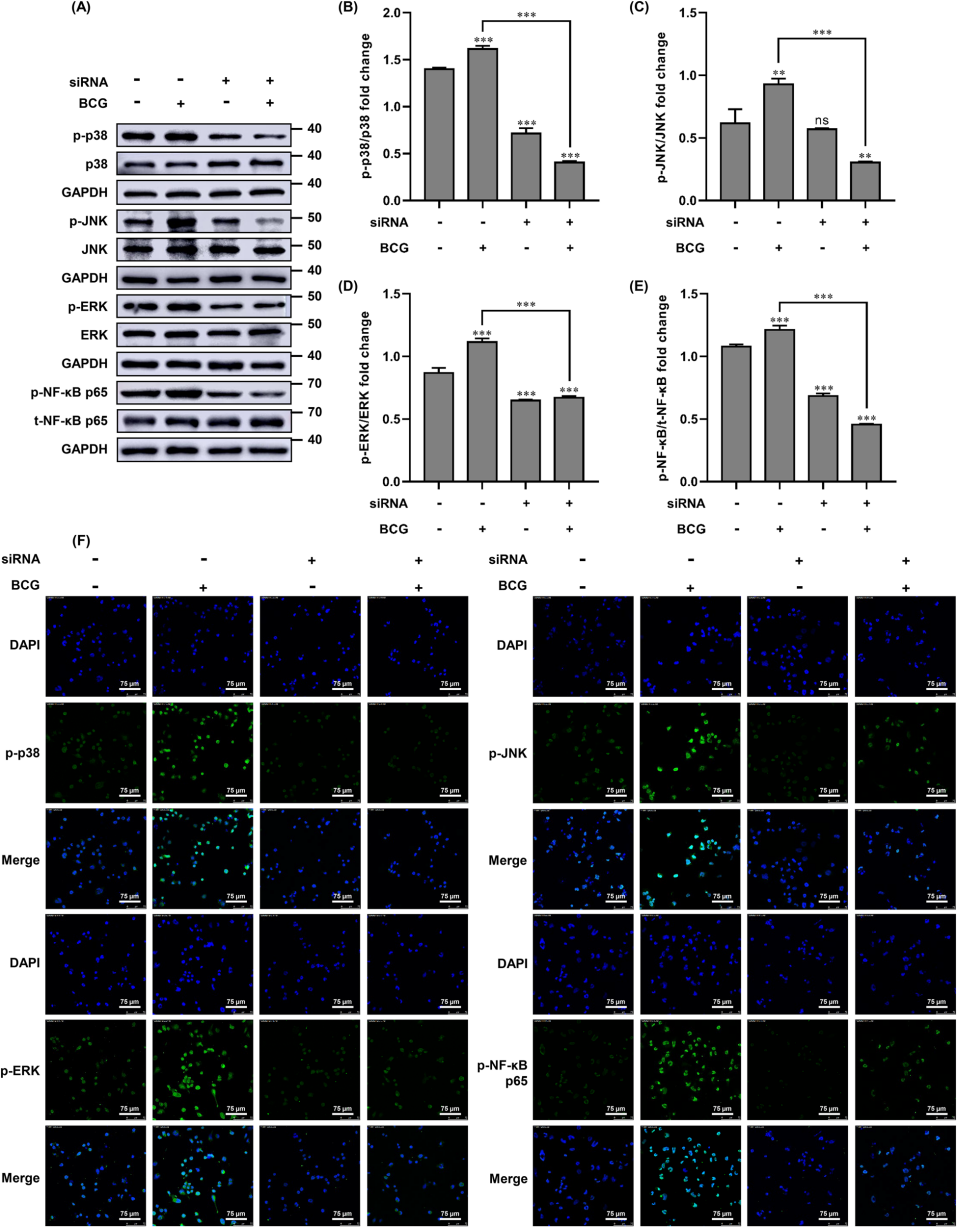
Effect of DUSP1 knockdown on MAPKs/NF-κB signaling pathway induced by BCG infection. (**A**) THP-1 cells were transfected with siRNA-NC (negative control) or siRNA-DUSP1 for 24 h, and then infected with BCG and cultured for 6 h. The expression of p-p38, p38, p-ERK, ERK, p-JNK, JNK, p-NF-κB p65 and t-NF-κB p65 were detected by Western blotting. (**B**–**E**) MAPKs/NF-κB signaling pathway results of grayscale analysis of key protein expression. (**F**) THP-1 cells were transfected with siRNA-NC (negative control) or siRNA-DUSP1 for 24 h, and then infected with BCG and cultured for 6 h. The expression levels of p-p38, p-ERK, p-JNK and p-NF-κB p65 were detected by immunofluorescence. Scale bar, 75 μm. All data are represented as mean ± SD (n = 3). (*p < 0.05, **p < 0.01, ***p < 0.001).

Based on our previous results showing that BCG can induce THP-1 macrophage apoptosis and promote TNF-α and IL-1β release, we hypothesized that DUSP1 might be involved in BCG-induced inflammatory response through the MAPKs/NF-κB signaling pathway. Therefore, we examined the expression levels of p-p38, p-ERK, p-JNK and p-NF-κB p65 in THP-1 cells by Western blotting. We found that treatment of THP-1 macrophages with siRNA-DUSP1 significantly reversed the BCG-induced phosphorylation of markers of p38, ERK, JNK, and NF-κB p65, but had no effect on the expression of p38, JNK, ERK, and NF-κB p65 (Fig. 5A–E). We then validated the results of Western blotting by immunofluorescence, and found that DUSP1 knockdown inhibits BCG-induced MAPKs/NF-κB signaling in THP-1 cells, which further relieved cellular apoptosis and inflammatory injury (Fig. 5F).

### In vivo study of the effects of BCG induced inflammation and the therapeutic potential of targeting DUSP1

**Figure 6:**
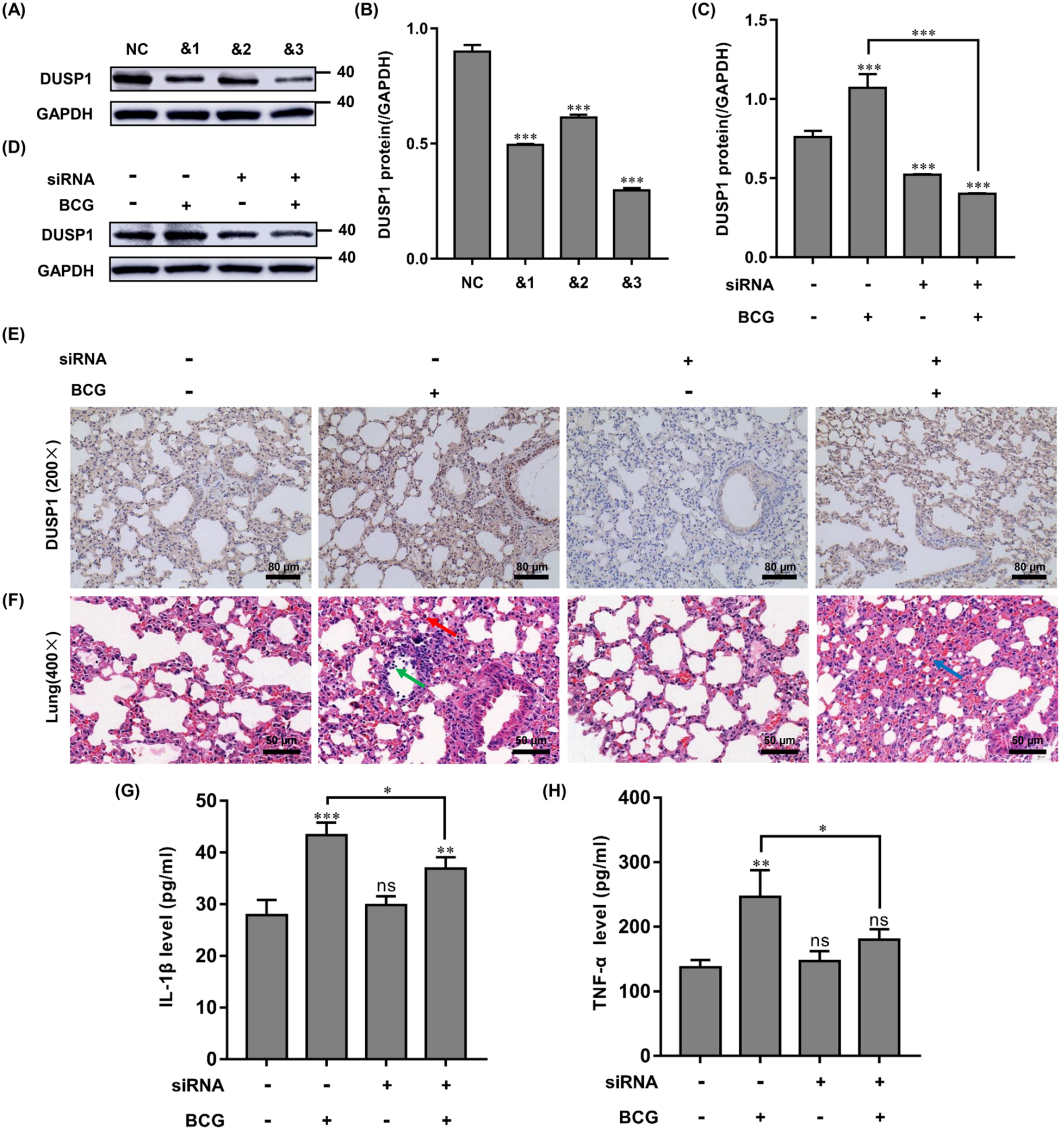
In vivo study of the effect of DUSP1 knockdown on BCG infection in a mouse model. (**A**, **B**) RAW264.7 cells were transfected with three different targeting siRNA-DUSP1 for 24 h, and the knockdown effect was verified by Western blotting. (**C**, **D**) RAW264.7 cells were transfected with siRNA-NC (negative control) or siRNA-DUSP1 for 24 h and then infected with BCG and cultured for 12 h. The expression of DUSP1 was detected using Western blotting. All data are represented as mean ± SD (n = 3). (**E**) C57BL/6J mice were infected with BCG by one-time direct airway perfusion (2 × 10^6^ CFU/only), and siRNA-DUSP1 was used to intervene in the lung DUSP1 expression of C57BL/6J mice by multiple airway perfusion into the mice using in vivo Advanced Transfection Reagent. 21 days after BCG infection, lung tissue was taken from mice and immunohistochemistry was performed to detect DUSP1 expression in lung tissues. Scale bar, 40 μm. (**F**) C57BL/6J mice were infected with BCG by direct airway infusion (2 × 10^6^ CFU/only) at one time, and siRNA-DUSP1 was infused into the lungs of C57BL/6J mice by multiple airway infusions using in vivo Advanced Transfection Reagent. 21 days after BCG infection, lung tissue was collected and analyzed for histopathological damage in the lungs of C57BL/6J mice by H&E staining. Scale bar, 50 μm. (**G**, **H**) The concentrations of TNF-α and IL-1β in the peripheral blood of mice in each treatment group were measured by ELISA, and the samples of mice in each treatment group were 6. (*p < 0.05, **p < 0.01, ***p < 0.001).

Three small interfering RNA sequences were designed and synthesized based on the murine-derived DUSP1 sequence, and siRNA-DUSP1 was transfected into RAW264.7 cells. The knockdown efficiency of the three small interfering RNAs was detected at the protein level. We found that compared with the negative control group, small interfering RNA No. 3 (&3) was the most efficient at knockdown, showing a significant downregulation of DUSP1 protein expression (Fig. 6A,B). To further verify the knockdown effect of siRNA-DUSP1, RAW264.7 cells were infected with BCG (MOI = 10) and cultured for 12 h, 24 h after siRNA-DUSP1 transfection. DUSP1 protein expression was significantly downregulated in the siRNA + BCG group compared to the BCG-alone group (Fig. 6C,D). Next, siRNA No. 3 (&3) was used to knockdown DUSP1 expression in C57BL/6J mouse lungs, by multiple administrations into the airway combined with in vivo Advanced Transfection Reagent. The mice were then infected with BCG by airway administration. After 21 days post-infection, the knockdown efficiency of siRNA-DUSP1 was analyzed by immunohistochemistry. We found that the expression level of DUSP1 in the lungs of siRNA-DUSP1 mice under BCG infection conditions was significantly reduced compared with the BCG-alone group (Fig. 6E), indicating that siRNA-DUSP1 can knockdown DUSP1 expression in the lung tissue of BCG-infected C57BL/6J mice. Based on this animal model, we further investigated the importance of DUSP1 in regulating BCG infection in vivo by H&E staining. We found that compared with control mice, the lung tissue of C57BL/6J mice in the BCG-alone group exhibited slight fibrosis, nodules, inflammatory infiltrates, and enlargement, in addition to slowed body weight gain and increased lung weight (data not shown). In contrast, the lung tissue of C57BL/6J mice given BCG infection combined with siRNA-DUSP1 were less damaged and displayed less inflammatory infiltrates (Fig. 6F). In addition, BCG infection also increased the expression of TNF-α and IL-1β, which was reversed by siRNA-DUSP1 (Fig. 6G,H). Overall, our in vivo study supported our hypothesis that siRNA-DUSP1 can reduce BCG-induced inflammatory injury.

### Effects of DUSP1 knockdown on protein expression of MAPKs/NF-κB signaling pathway in BCG-infected C57BL/6J mice lung tissue

**Figure 7:**
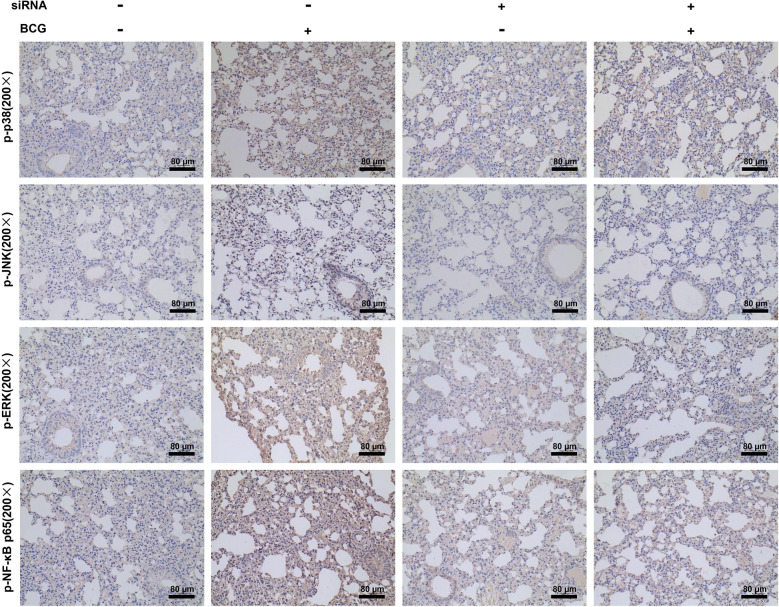
Immunohistochemical analysis of MAPKs/NF-κB signaling pathway proteins in the lungs of DUSP1 knockdown combined with BCG infection in C57BL/6J mice. C57BL/6J mice were infected with BCG (2 × 10^6^ CFU/only) by a single direct airway infusion, and siRNA-DUSP1 was administered to downregulate DUSP1 expression in the lungs of C57BL/6J mice by multiple airway infusions into the mice using in vivo Advanced Transfection Reagent. After 21 days of BCG infection, lung tissues were taken from mice and immunohistochemically examined for p-p38, p-ERK, p-JNK and p-NF-κB p65 expression in lung tissue. The samples of mice in each treatment group were 6. Scale bar, 40 μm. (*p < 0.05, **p < 0.01, ***p < 0.001).

In vitro studies have shown that DUSP1 interference inhibits the activation of MAPKs/NF-κB signaling pathway after BCG infection. We applied immunohistochemistry to detect the expression of p-p38, p-ERK, p-JNK and p-NF-κB p65 proteins in mouse lung tissues, and found that compared with the control group, lung tissues of C57BL/6J mice in the BCG group showed higher expression of p-p38, p-ERK, p-JNK and p-NF-κB p65, while knockdown of DUSP1 significantly downregulated the expression of p-p38, p-ERK, p-JNK and p-NF-κB p65 (Fig. 7). These results further supported the hypothesis that interfering with DUSP1 inhibited the BCG-induced inflammatory response through MAPKs/NF-κB signaling pathway at the in vivo level.

## Discussion

Macrophages are the main host cells of Mtb and their apoptosis, necrosis, autophagy and inflammation are essential components of the host defense against Mtb^22^. BCG can induce macrophage apoptosis and inflammation by activation of MAPKs/NF-κB signaling pathway^23–25^, and DUSP1 is a dual-specificity phosphatase that regulates MAPKs activity. Therefore, in this study, we investigated the role of DUSP1 in BCG-induced apoptosis and inflammation and its possible mechanism in THP-1 cells, which can be used to help to discover new therapies for TB.

Studies have shown that DUSP1 expression can be upregulated in response to a variety of stimuli, including growth factors^26^, LPS^27^, p53^28^, heat shock^29^ and UV damage^30^. Our study found that BCG infection induced cell apoptosis in THP-1 cells and promoted the expression of DUSP1, suggesting that DUSP1 may be involved in regulation of BCG-induced macrophage apoptosis. Recent studies have revealed that DUSP1 plays an important negative regulatory role in keratinocyte apoptosis^17^ and antitumor immune response^31^. Interestingly, our results found that knockdown of DUSP1 expression caused a significant reduction in the expression levels of cleaved-Caspase3 and cleaved-PARP1, suggesting that DUSP1 has a positive regulatory effect on BCG-induced cell apoptosis. It has also been found that DUSP1 has a pro-apoptotic function independent of JNK and p38 during Sendai virus infection^32^. The interaction of JNK with JNK interacting protein 1 (JIP1) was found to have a protective effect on DUSP1 expression, which prevented the negative regulation of cytokine responses by DUSP1^32^. DUSP1 inhibition was shown to significantly reduce the rate of apoptosis in bovine oocytes^33^, and knockdown of DUSP1 in pancreatic cancer cells also attenuated the tumorigenicity in a nude mouse model^34^. These various studies revealed that DUSP1 promotes apoptosis in addition to its usual phosphatase activity.

To better understand the effect of DUSP1 on BCG-induced apoptosis, we detected cleaved-Caspase9, Bax, cytochrome C and Apaf-1, all of which are key proteins of the intrinsic apoptotic pathway^21^, We detected decreased expression of key proteins of the intrinsic apoptotic pathway in DUSP1 knockdown THP-1 cells. In contrast, the intrinsic pathway of apoptosis is regulated by the Bcl-2 family. It is believed that Bcl2-Bax heterodimer formation and stoichiometry is important to maintain mitochondrial function^20^. When cells are stimulated by external pro-apoptotic signals, Bax proteins form homodimers and stimulate the release of cytochrome C from mitochondria, which activates a downstream caspase cascade response^20,35^. In agreement with these findings, in our study, DUSP1 knockdown increased expression of the anti-apoptotic protein Bcl-2 during BCG infection. Therefore, our results indicated that DUSP1 knockdown inhibited BCG-induced apoptosis via the intrinsic apoptotic pathway.

MAPKs/NF-κB is considered to be an important pathway that regulates inflammation and apoptosis, and host-derived TNF-α and IL-1β can activate the MAPKs/NF-κB pathway in macrophages, which in turn activates the production of pro-inflammatory cytokines, including TNF-α and IL-1β themselves^16^. In this study, we found that BCG could induce the activation of MAPKs and NF-κB. Knockdown of DUSP1 reduced activation of MAPKs and NF-κB and BCG-induced release of TNF-α and IL-1β. Recent studies have confirmed that knockdown of DUSP9^36^, DUSP12^37^, DUSP14^38^ and DUSP26^39^ exacerbated the inflammatory response in the liver. However, in a mouse model of obesity caused by a high-fat diet, DUSP6 exhibited the opposite effect^40,41^. Oxidized low-density lipoprotein (ox-LDL) is commonly used to induce macrophage foaminess, but also induces DUSP10 expression in macrophages. A study by Luo, et al., found that knockdown of DUSP10 inhibited NF-κB phosphorylation but promoted macrophage foaminess^42^. Taken together, these results demonstrate that apoptosis and inflammation are not independent biological processes, but instead are both regulated by complex cellular signaling. The study of apoptosis and inflammatory response induced by BCG infection in our study also plays a role in fully understanding this complex process.

Our in vivo data showed that DUSP1 knockdown significantly reduced BCG-induced lung injury and the release of IL-1β and TNF-α in C57BL/6J mice, which is consistent with our in vitro findings. Furthermore, the in vivo study showed a potential role of DUSP1 as a regulator of apoptosis and inflammation in mice infected with BCG. To further support this finding, lipopolysaccharide (LPS) was used as a positive control, and we found that DUSP1 played a negative regulatory role in LPS-induced cleaved-Caspase3 expression (Supplementary Fig. 1). Thus far, all experimental results from this study confirmed the positive role played by DUSP1 in regulating BCG-induced apoptosis and inflammation. Other studies have demonstrated that DUSP2^43^ and DUSP6^44^ also play a positive regulatory role in the bacterial infection response of macrophages, most likely because the expression of DUSPs is not only transcriptionally regulated by MAPK signaling, but also at the protein level, where MAPKs directly influence the stability of DUSPs through phosphorylation. Sakaue, et al. found that ERK1/2 increased the stability of DUSP1 through direct phosphorylation, leading to a decrease in ubiquitination and proteasomal degradation ^45^. Stimulation in KO mast cells and macrophages showed that DUSP2 inhibited JNK phosphorylation, while use of a JNK inhibitor restored ERK1/2 phosphorylation and increased DUSP2 stability ^43^.

In summary, the present study demonstrated that DUSP1 was induced in THP-1 macrophages in response to BCG infection, and that DUSP1 acts as a regulator of MAPKs/NF-κB signaling pathway. It also plays an important role in regulating BCG-induced macrophage apoptosis and inflammatory response, suggesting that DUSP1 is a potential candidate for anti-TB drug discovery that warrants further investigation. Furthermore, BCG induced the expression of DUSP1 and simultaneously activated MAPKs. There are still unknown pathways or kinases downstream of DUSP1 that in turn inhibit the activation of MAPKs, ultimately leading to the production of pro-apoptotic proteins and pro-inflammatory cytokines that trigger macrophage apoptosis and inflammatory responses. Future studies are needed to screen proteins that interact with DUSP1 after BCG infection, which will further support our hypothesis and promote DUSP1 as a candidate for future clinical applications.

## Materials and methods

### Cell culture

Human myeloid leukemia mononuclear cells THP-1 and mouse macrophage-like (RAW264.7) cell were obtained from the Cell Bank of the Chinese Academy of Sciences (Shanghai, China). THP-1 cells were cultured in RPMI 1640 medium (Gibco, USA) supplemented with 10% fetal bovine serum (Ausbian, USA) and 1% penicillin/streptomycin (Solarbio, China). RAW264.7 cells were cultured in full DMEM (Gibco, USA) containing 10% FBS. Culturing was done at 37 °C and 5% CO_2_ in a constant temperature and humidity incubator.

### BCG culture and infection

Bacillus Calmette-Guérin (BCG) was purchased from the Chinese Centre for Disease Control and Prevention (Beijing, China). Cultures were grown in Middlebrooks 7H9 broth (Becton Dickinson & Company, USA) containing 0.5% glycerol, 0.05% Tween-80 and 10% OADC. Cultures were incubated in a constant temperature incubator at 37 °C, and the cells were collected by centrifugation when the culture had reached the appropriate density (OD600 = 0.5). Prior to infection, THP-1 cells grown to log phase were seeded into six-well plates with 1 × 10^6^ cells per well. THP-1 cells were cultured for 24 h in medium containing 50 ng/mL PMA (Sigma-Aldrich, USA), which was used to induce THP-1 monocyte differentiation into macrophages. Adherent cells were then infected with BCG at an MOI of 10.

### Construction and transfection of DUSP1 small interfering RNAs

Three small interfering RNA sequences (Table 1) were designed against the sequence of the human DUSP1 coding region and synthesized by Genepharma (Shanghai, China). THP-1 cells were induced in a 6-well plate using PMA, seeded at a density of 1 × 10^6^ cells per well before transfection with the small interfering RNA (siRNA-DUSP1), for those groups without adding siRNA-DUSP1, siRNA-NC was added as the negative control. Transfections were performed using Lipofectamine™ RNAi MAX reagent (Invitrogen, USA) according to the manufacturer's protocol and the fluorescence intensity was observed by fluorescence microscopy 24 h post-transfection.

**Table 1:** The sequence of small interfere RNA.

siRNA	Sense (5’-3’)	Antisense (5’-3’)
#1	GCUCCUUCUUCGCUUUCAATT	UUGAAAGCGAAGAAGGAGCTT
#2	CUCCCAACUUCAGCUUCAUTT	AUGAAGCUGAAGUUGGGAGTT
#3	CGAGGCCAUUGACUUCAUATT	UAUGAAGUCAAUGGCCUCGTT
NC	UUCUCCGAACGUGUCACGUTT	ACGUGACACGUUCGGAGAATT

### RNA isolation and real-time PCR

Total RNA was extracted from THP-1 cells using the Total RNA Extraction Kit (TIANGEN, China) following the manufacturer's protocol. The first strand was synthesized from 1 μg of total RNA using the Reverse Transcription System Kit (Takara, China) and RT-qPCR was performed using SYBR Premix ExTaq II (Takara, China) in a total volume of 20 μL. Sequences of primers using in RT-qPCR are listed in Table 2. Expression levels are normalized to GAPDH, with each assay performed in triplicate. Relative fold changes in messenger RNA expression were calculated using the 2^-ΔΔCt^ method.

**Table 2:** Gene specific primers for RT-qPCR.

Gene	Primers	Primers sequence (5’-3’)
*GAPDH*	Forward	TGACATCAAGAAGGTGGTGAAGCAG
	Reverse	GTGTCGCTGTTGAAGTCAGAGGAG
*DUSP1*	Forward	ACAACCACAAGGCAGACATCAGC
	Reverse	CCTCATAAGGTAAGCAAGGCAGATGG
*TNF-α*	Forward	CAATGGCGTGGAGCTGAGAGATAAC
	Reverse	TTGAAGAGGACCTGGGAGTAGATGAG
*IL-1β*	Forward	TGATGGCTTATTACAGTGGCAATGAGG
	Reverse	TGTAGTGGTGGTCGGAGATTCGTAG

### Western Blotting

Total protein was extracted from THP-1 cells using a total protein extraction kit (KeyGEN BioTECH, China), following which cell lysate was spun at 12,000 rpm. The supernatant was then collected and protein concentration was determined via BCA assay (ThermoFisher, USA). The protein samples were then heated at 100 ℃ in 1 × protein loading buffer (Transgene, China) for 5 min and separated via 10% SDS-PAGE. Blots were then wet transferred to PVDF membranes, blocked with 5% skimmed milk for 1 h at room temperature and phosphorylated antibodies were blocked with 3% BSA. Samples were then incubated overnight after addition of detection antibodies at the following dilution factor: DUSP1 (1:1000); ERK (1:1000); p-ERK (1:1000); p-NF-κB p65 (1:1000; all from Affinity, China); Bcl-2 (1:2000); Bax (1:2000); Cytochrome C (1:1000); Apaf-1 (1:1000); Cleaved-PARP1 (1:2000); Cleaved-caspase3 (1:2000); Cleaved-caspase9 (1:1000; all from Abcam, UK); p38 (1:1000); p-p38 (1:1000); JNK (1:1000); p-JNK (1:1000; all from ABclonal, China); and GAPDH (1:2000; Abmart, China). Membranes were then incubated with horseradish peroxidase (HRP)-coupled IgG for 1 h at room temperature (1:5000; Abmart, China). After washing with TBST, immunoreactive bands were observed using the ECL kit (Advansta Inc., USA) and scanned for protein bands using Amersham ImageQuant 800 (Cytiva, Japan). Finally, protein bands were then quantified using ImageJ software.

### Detection of apoptosis by flow cytometry

THP-1 cells (1 × 10^6^ cells/well) were seeded in 6-well plates, transfected with siRNA-DUSP1, incubated for 24 h, and infected with BCG (MOI = 10) and incubated for another 6 h. Apoptosis was detected by FACS using an Annexin V/propidium iodide (PI) kit (KeyGEN BioTECH, China). Cells were then harvested and washed twice with PBS, then resuspended in 500 μL binding buffer. 5 μL of Annexin V staining solution and PI staining solution were added to each sample and incubated for 15 min at room temperature protected from light. After centrifugation at 1000 rpm for 5 min, the cell pellets were resuspended in 500 μL binding buffer and the samples were analyzed at 535 nm (PI) and 488 nm (Annexin V) using a FACS Canto™ II cytometer (BD, USA).

### Detection of mitochondrial membrane potential (MMP) by flow cytometry

THP-1 cells (1 × 10^6^ cells/well) were seeded in 6-well plates, transfected with siRNA-DUSP1, incubated for 24 h, and then infected with BCG (MOI = 10) and incubated for another 6 h. MMP changes were analyzed with the JC-1 kit (ThermoFisher Scientific, USA) and FACS. Briefly, cells in different groups were harvested after treatment. A separate positive control group is also required. Cells in the positive control group were treated with CCCP (20 μM) for 30 min. Then cells in all groups were washed three times with PBS, and then resuspended in 500 μL of PBS. Cell suspensions were then stained with 200 μM JC-1 (5 μL, final concentration = 2 μM) at 37 °C and 5% CO_2_ for 30 min. Each sample was detected at 488 nm using a FACS Canto™ II cytometer (BD, USA). Analysis was then performed with FlowJo® software.

### Immunofluorescence assay

THP-1 cells (1 × 10^5^ cells/well) were seeded in 12-well plates with cell coverslips, transfected with siRNA-DUSP1, and cultured for 24 h post-transfection. Cells were infected with BCG (MOI = 10) and incubated for another 6 h. The cells were then washed 3 times with PBS and fixed with 4% paraformaldehyde for 20 min. The cells were washed and permeabilized with 0.5% TritonX-100 for 20 min, then blocked with 3% BSA for 1 h at room temperature. Antibodies were diluted in 3% BSA at dilution factors of DUSP1 (1:200), Cleaved-Caspase3 (1:200), Cleaved-PARP1 (1:200), p-p38 (1:100), p-JNK (1:100), p-ERK (1:200), and p-NF-κB p65 (1:200). 500 μL of the primary antibody was added to each well and incubated overnight at 4 °C. After washing three times with PBS, cells were incubated with fluorescein-coupled secondary antibody (1:1000) in PBS for 1 h at 37 °C, protected from light. Finally, cells were stained with a fluorescent blocker containing DAPI (ORIGENE, China) blocked, and images were acquired with a Leica TCS SP2 A0BS confocal system and processed on Leica confocal software (Leica, Germany).

### Enzyme-linked immunosorbent assay

The ELISA kits for IL-1β and TNF-α were obtained from Thrive Biotechnology (Shanghai, China). Peripheral blood was collected from mice in different treatment groups and the levels of inflammatory cytokines TNF-α and IL-1β were measured by ELISA. The experiments were performed according to the manufacturer's instructions, and the experiments were repeated three times and the mean values were taken.

### Transmission electron microscopy

THP-1 cells were washed with PBS, fixed with 3% glutaraldehyde, refixed with 1% osmium tetroxide, dehydrated step by step in acetone, and embedded using Ep812 (EMCN, China). Ultra-thin sections were then made with a diamond knife, sections were stained with uranyl acetate (EMCN, China) and lead citrate (EMCN, China) and observed by JEM-1400FLASH transmission electron microscopy (JEOL, Japan).

### Animal experiments

C57BL/6J mice were purchased from Chengdu Dashuo Experimental Animal Co., Ltd. (Chengdu, China). The mice were housed in a special pathogen-free room with food and water ad libitum and regular 12:12 light–dark cycle. Three siRNAs targeting mouse DUSP1 (Table 3) were transfected into RAW264.7 cells using Lipofectamine™ RNAi MAX Reagent (Invitrogen, USA) according to the manufacturer's protocol to screen for the best siRNA-DUSP1. In vivo experiments were performed using Advanced Transfection Reagent (ZETA, USA) to interfere with lung DUSP1 expression in C57BL/6J mice by multiple airway infusions. 24 male C57BL/6J mice (8 weeks old) were used for in vivo experiments. There were 4 treatment groups (6 animals in each group), siRNA-NC group, siRNA-NC + BCG infection group, siRNA-DUSP1 group and siRNA-DUSP1 + BCG infection group. The mice were weighed and recorded before drug treatment. BCG was diluted to 2 × 10^6^ CFU/100 µL suspension using saline. siRNA-DUSP1 was prepared to 1 OD/50 µL using nuclease-free water, siRNA-DUSP1 was prepared to 20 µg/100 µL working solution using saline, and saline solution alone was used as a negative control (100 µL). Diluted BCG suspension or saline was intranasally instilled into the respiratory tract of the isoflurane-anesthetized mice using a 100 µL pipette as previously described^46^. siRNA-DUSP1 working solution was administered in the same way one day after BCG infection, and mice were treated every 6 days. Mice were sacrificed on day 21, and the concentrations of TNF-α and IL-1β in peripheral blood were determined at terminal timepoint using ELISA. Lung tissues were collected, fixed and stained with haematoxylin and eosin (H&E) as previously described, before performing immunohistochemical analysis, imaging of the sections using a microscopic camera system, and calculation of the percentage of positive area per image using the Halo data analysis system.

**Table 3:** The sequence of small interfere RNA.

siRNA	Sense (5’-3’)	Antisense(5’-3’)
&1	GUGCCCUGAACUACCUUAATT	UUAAGGUAGUUCAGGGCACTT
&2	CCACUCAAGUCUUCUUUCUTT	AGAAAGAAGACUUGAGUGGTT
&3	GCUCCUUCUUCGCUUUCAATT	UUGAAAGCGAAGAAGGAGCTT
NC	UUCUCCGAACGUGUCACGUTT	ACGUGACACGUUCGGAGAATT

### Ethics statement

All animal experiments complied with the ARRIVE guidelines and were carried out following US National Institutes of Health Guidelines for the Use and Care of Laboratory Animals (NI Publications No. 85–23, revised 1985) and Use of Laboratory Animals and the Chinese Legislation on Laboratory Animals. The animal experimental protocol in this study complied with the Animal Management Rules of the Chinese Ministry of Health (document no. 55, 2001) and was approved by the Research Ethics Committee of the Institutional Animal Care and Use Committee of Ethics Committee of Sichuan Lilaisinuo (Experiment License: LLSN-2022002).

### Statistical analysis

Statistical tests were conducted with GraphPad Prism v8.0 (GraphPad Software, USA). All results were described as mean ± SD and were derived from 3 biological replicates. The difference between the 2 groups was compared by one-way ANOVA.

## Supplementary Information

Supplementary Information 1.

Supplementary Information 2.

Supplementary Information 3.

Supplementary Information 4.

Supplementary Information 5.

Supplementary Information 6.

Supplementary Information 7.

Supplementary Information 8.

## Data Availability

All data generated or analyzed during this study are included in this published article (and its Supplementary Information files).
