# Supplementary material for: DUSP1 mediates BCG induced apoptosis and inflammatory response in THP-1 cells via MAPKs/NF-κB signaling pathway
Source: Sci Rep. 2023 Feb 14;13:2606. doi: 10.1038/s41598-023-29900-6 (PMC9926451; doi:10.1038/s41598-023-29900-6)
Supplement: Supplementary file 1 — Supplementary Information 1. [file 41598_2023_29900_MOESM1_ESM.pdf]

Figure 1(A)

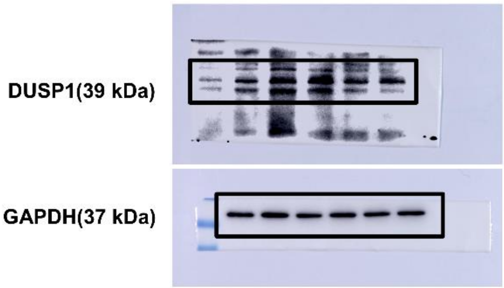

Figure 1(B)

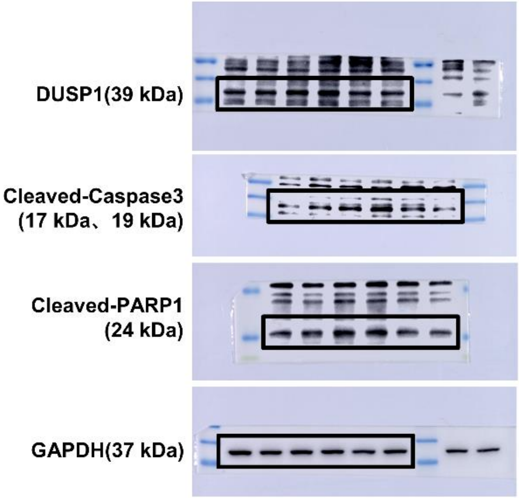

Gels and blots which are shown cropped in Figure 1(A), (B). Note: The blot was cut into multiple strips following the protein transfer. The edges of the PVDF membrane are visible. Here, bands containing the following regions were separately imaged: DUSP1(30-55 kDa), GAPDH (30-55 kDa), Cleaved-Caspase3 (10-40 kDa) and Cleaved-PARP1(15-40 kDa).
