# Supplementary material for: DUSP1 mediates BCG induced apoptosis and inflammatory response in THP-1 cells via MAPKs/NF-κB signaling pathway
Source: Sci Rep. 2023 Feb 14;13:2606. doi: 10.1038/s41598-023-29900-6 (PMC9926451; doi:10.1038/s41598-023-29900-6)
Supplement: Supplementary file 2 — Supplementary Information 2. [file 41598_2023_29900_MOESM2_ESM.pdf]

Figure 2(A)

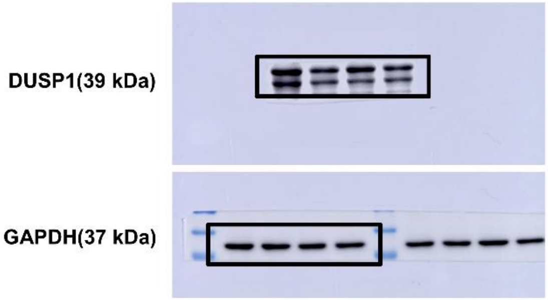

Figure 2(C)

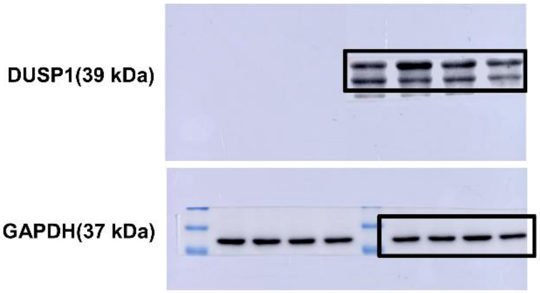

Gels and blots which are shown cropped in Figure 2(A), (C). Note: The blot was cut into multiple strips following the protein transfer. The edges of the PVDF membrane are visible. Here, bands containing the 30-50 kDa and 30-55 kDa regions were imaged, respectively.
