# Supplementary material for: DUSP1 mediates BCG induced apoptosis and inflammatory response in THP-1 cells via MAPKs/NF-κB signaling pathway
Source: Sci Rep. 2023 Feb 14;13:2606. doi: 10.1038/s41598-023-29900-6 (PMC9926451; doi:10.1038/s41598-023-29900-6)
Supplement: Supplementary file 4 — Supplementary Information 4. [file 41598_2023_29900_MOESM4_ESM.pdf]

Figure 4(A)

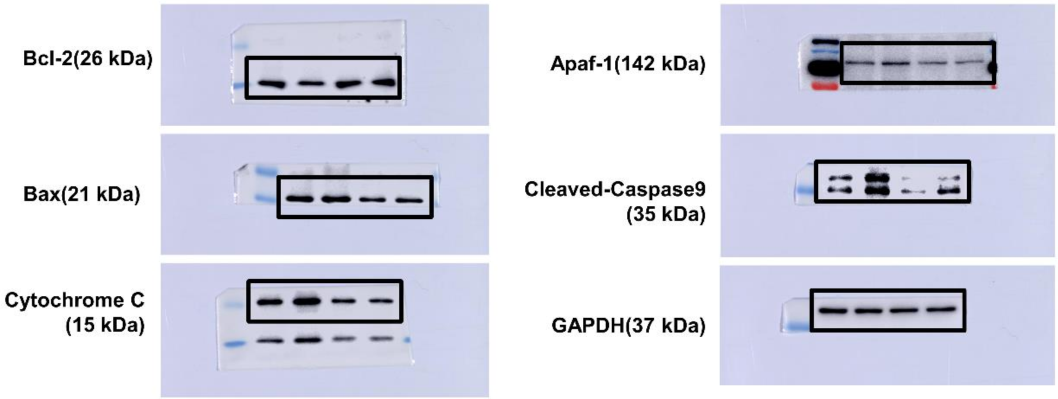

Gels and blots which are shown cropped in Figure 4(A). Note: The blot was cut into multiple strips following the protein transfer. The edges of the PVDF membrane are visible. Here, bands containing the following regions were separately imaged: Bcl-2(15-40 kDa), Bax (10-35 kDa), Cytochrome C (10-25 kDa), Apaf-1(70-180 kDa), Cleaved-Caspase9 (30-45 kDa) and GAPDH (30-45 kDa).
