# Supplementary material for: DUSP1 mediates BCG induced apoptosis and inflammatory response in THP-1 cells via MAPKs/NF-κB signaling pathway
Source: Sci Rep. 2023 Feb 14;13:2606. doi: 10.1038/s41598-023-29900-6 (PMC9926451; doi:10.1038/s41598-023-29900-6)
Supplement: Supplementary file 5 — Supplementary Information 5. [file 41598_2023_29900_MOESM5_ESM.pdf]

Figure 5(A)

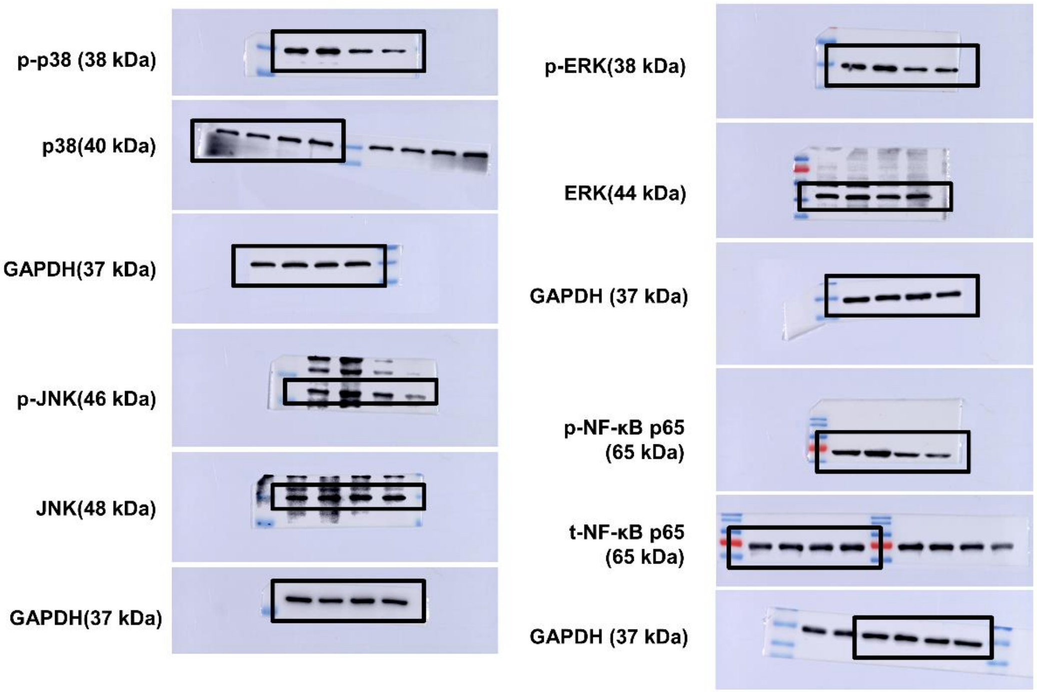

Gels and blots which are shown cropped in Figure 5(A). Note: The blot was cut into multiple strips following the protein transfer. The edges of the PVDF membrane are visible. Here, bands containing the following regions were separately imaged: p-p38(30-55 kDa), p38(30-55 kDa), p-JNK (30-60 kDa), JNK (30-60 kDa), p-ERK (30-60 kDa), ERK (30-110 kDa) and GAPDH (30-50 kDa).
