# Supplementary figures and images for: DUSP1 mediates BCG induced apoptosis and inflammatory response in THP-1 cells via MAPKs/NF-κB signaling pathway
Source: Sci Rep. 2023 Feb 14;13:2606. doi: 10.1038/s41598-023-29900-6 (PMC9926451; doi:10.1038/s41598-023-29900-6)

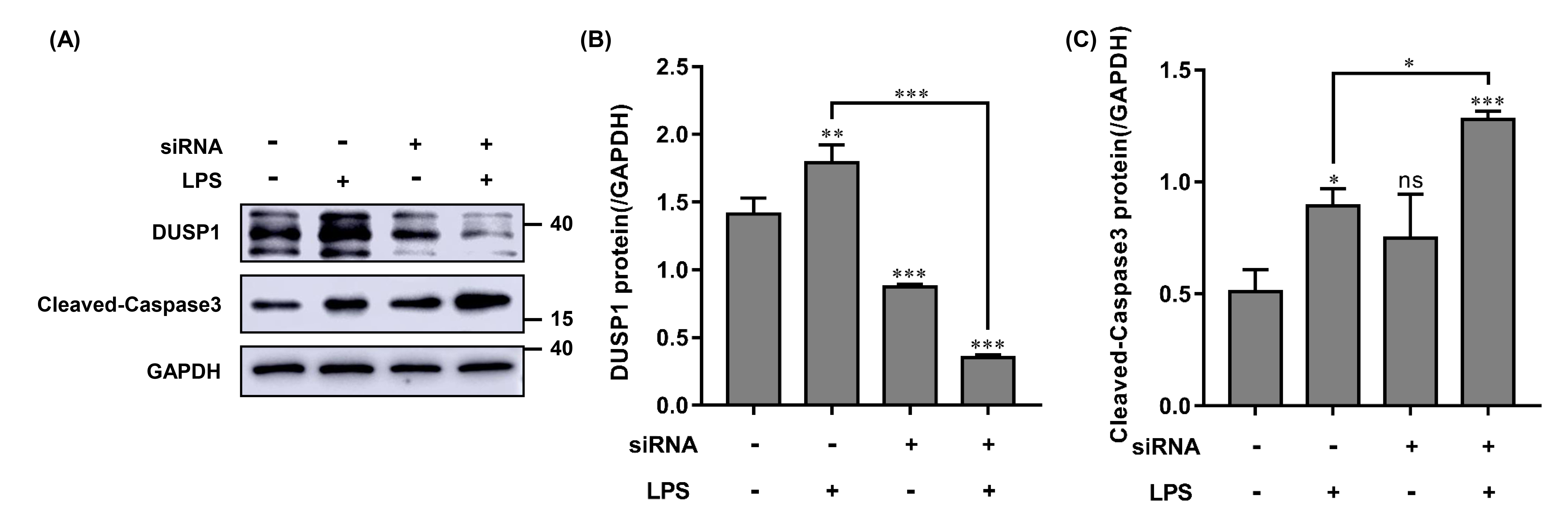

Supplement: Supplementary file 8 — Supplementary Information 8. [file 41598_2023_29900_MOESM8_ESM.tif]
